# Effects of a novel acetaminophen analog on cardiorespiratory compensatory responses and survival in a male rat model of traumatic hemorrhage

**DOI:** 10.14814/phy2.70619

**Published:** 2025-11-16

**Authors:** Miryam M. Pando, Kathy L. Ryan, Mariam L. Calderon, Cassandra M. Rodriguez, Brian S. Connor, Samantha L. Perez, Kevin D. Bunker, Chad D. Hopkins, Harold G. Klemcke, Lonnie E. Grantham, Carmen Hinojosa‐Laborde

**Affiliations:** ^1^ U.S. Army Institute of Surgical Research, JBSA Fort Sam Houston San Antonio Texas USA; ^2^ Kalyra Pharmaceuticals San Diego California USA; ^3^ Present address: 59th Medical Wing, JBSA‐Lackland San Antonio Texas USA; ^4^ Present address: Lincoln Memorial University College of Veterinary Medicine Harrogate Tennessee USA; ^5^ Present address: Kthera, LLC San Diego California USA; ^6^ Present address: Integrated Pharma Consulting, LLC Fishers Indiana USA; ^7^ Present address: U.S. Army Institute of Surgical Research, JBSA Fort Sam Houston San Antonio Texas USA

**Keywords:** acetaminophen, analgesia, hemorrhage, pain management, trauma

## Abstract

When pain is associated with traumatic hemorrhage, medics must be concerned about secondary effects of analgesics on cardiorespiratory systems. A novel analog of acetaminophen, D‐112, was developed to circumvent liver toxicity and improve analgesic efficacy. D‐112 causes dose‐related inhibition of formalin‐induced licking. The objective of this study was to test the effects of D‐112 on survival and cardiorespiratory variables following hemorrhage and extremity trauma (ET). We hypothesized that D‐112 would significantly change cardiorespiratory responses to HEM and thereby decrease survival. Male rats received either vehicle (lactated Ringer's) or D‐112 (50 mg/kg) after conscious hemorrhage of either 37% (*n* = 10, vehicle and D‐112) or 50% (*n* = 8, vehicle; *n* = 11, D‐112) of blood volume following ET, which consisted of soft tissue injury and fibula fracture. Rats were observed for 4 h after the start of hemorrhage. Neither survival times (37% hemorrhage: *p* = 0.474; 50% hemorrhage: *p* = 0.306) nor survival curves (37% hemorrhage: *p* = 0.146; 50% hemorrhage: *p* = 0.280) differed between treatments. Mean arterial pressure did not differ between treatments (37% hemorrhage: *p* = 0.742; 50% hemorrhage: *p* = 0.521). D‐112 transiently elevated minute ventilation (*p* < 0.001) after both hemorrhages. D‐112 does not alter cardiorespiratory responses to the point of depressing survival, suggesting that D‐112 could be an appropriate analgesic following traumatic hemorrhage.

## 1 INTRODUCTION

Pain is often associated with physical trauma or other stressors encountered during civilian accidents or military combat (Aldington et al., 2011). Relief of pain is a major emphasis of the United States Army, and attempts at this relief begin early on the battlefield (Clifford et al., 2014). A recent consensus of experts on battlefield pain management identified that (1) research on alternative nonopioid analgesics is needed and (2) research should prioritize the most common battlefield patterns of injury (Stark et al., 2022). Multiple analgesics are used for acute pain relief in both military and civilian settings. However, a medical provider must also be concerned about secondary effects of analgesics, especially effects on the cardiovascular and respiratory systems when the pain of trauma is associated with compromised physiological states such as following hemorrhage. Thus, there is a continued need to develop novel analgesics that provide safe and effective pain management at the point of injury without cardiovascular or respiratory suppression or cognitive impairment.

Currently, acetaminophen (APAP) is recommended by the Tactical Combat Casualty Care Guidelines for use when pain is mild to moderate, and the individual may still participate in combat (Butler Jr. et al., 2014). A 1000 mg dose of APAP has maximum analgesic activity in human adults. However, APAP at high doses (>250 mg/kg) can lead to liver damage and subsequent liver failure that is often fatal (Heldring et al., 2022; Prescott, 1983). APAP overdose poses a significant issue within the military sector (Clark & Taubman, 2016; Taylor et al., 2012) and the civilian population (Bari & Fontana, 2014) with both intentional and unintentional exposures accounting for thousands of hospitalizations and hundreds of deaths annually (Agrawal et al., 2025). A novel class of analgesics developed by Kalyra Pharmaceuticals involves analogs of APAP that have been modified to circumvent liver toxicity and improve the analgesic effects of APAP. Kalyra Pharmaceuticals replaced structural components of APAP and synthesized several structurally related APAP analogs that eliminate the formation of a highly reactive metabolite, N‐acetyl‐*p*‐benzoquinone imine (NAPQI), associated with liver toxicity (Reddoch‐Cardenas et al., 2021). Importantly, these analogs also have significantly increased analgesic potency and provide a longer half‐life than APAP. These analogs have the potential to replace opioids for severe pain without the accompanying side effects. In this study, we focus on one such APAP analog, D‐112.

Literature is unavailable for D‐112 with reference to effects on cardiorespiratory function; however, studies have been conducted with APAP itself. There is no documented evidence for effects of APAP on respiration, except at exceptionally high, toxic doses wherein APAP depresses respiration (Bertolini et al., 2006; Shah et al., 2011). In contrast, APAP does affect cardiovascular function. When reviewed over multiple studies with diverse patient populations, intravenous APAP was shown to produce hypotension (decreased mean arterial blood pressure (MAP)) that was clinically relevant (Maxwell et al., 2019). On the contrary, in patients with coronary artery disease, APAP very modestly, but significantly, increased MAP (Sudano et al., 2010). In rats, APAP was cardio‐protective during cardiac ischemia (Zhu et al., 2006) and after ischemia–reperfusion injury (Geldi et al., 2018). In dogs, APAP improved cardiac rhythmicity after ischemia–reperfusion (Merrill et al., 2007). In rabbits and sheep, APAP neither exacerbated nor improved cardiac function after ischemia–reperfusion, but increased MAP prior to ischemia (Leshnower et al., 2006). Hence, based on the divergent results associated with APAP itself, there is a possibility for cardiovascular effects of APAP analogs. Therefore, we tested the effects of the novel APAP analog, D‐112, on the compensatory cardiorespiratory responses and on survivability following hemorrhage and trauma. We hypothesized that D‐112 would change cardiovascular and respiratory responses to HEM and thereby decrease survival.

## 2 METHODS

### 2.1 D‐112 formalin test at Kalyra pharmaceuticals

Male Sprague–Dawley rats (BioLasco Taiwan under Charles River Laboratories Licensee) weighing 200 ± 20 g were acclimatized to the laboratory conditions at least 1 week before experimentation. The rats were housed at a temperature of 20–24°C with 12 h light/dark cycles. Standard lab food (MF (Basic Feed) rodent chow (Oriental Yeast Co., Ltd., Tokyo, Japan)) and autoclaved tap water were provided ad libitum. Research was conducted in compliance with the Animal Welfare Act, the implementing Animal Welfare Regulations, and the principles of the Guide for the Care and Use of Laboratory Animals: Eighth Edition, National Research Council at Eurofins Panlabs Taiwan (Taipei, Taiwan). The procedure for the formalin test, a commonly used test to measure pain and its inhibition by putative analgesics, closely followed that described by Dubuisson et al. and others (Abbott et al., 1995; Dubuisson & Dennis, 1977; Xiaoqiang et al., 2024). The acute phase of the formalin test is caused primarily by C‐fiber activation due to peripheral stimulus while the late phase is a combination of an acute inflammatory reaction in the peripheral tissue and functional changes in the dorsal horn of the spinal cord (Abbott et al., 1995; Hoffmann et al., 2022; McNamara et al., 2007; Tjolsen et al., 1992). Vehicle (50 mM citrate buffer, pH 5.5) or drug (APAP at 300 mg/kg, D‐112 at 1, 3, 10, or 30 mg/kg) was administered intravenously to separate groups of rats (*n* = 8/group) 10 min prior to sub‐plantar injection of formalin (0.05 mL, 2% solution). The hind paw licking time was recorded at 5‐min intervals for 35 min after formalin challenge as a measure of the analgesic activity of the test compound. The acute phase is defined as 0–5 min and the late phase is defined as 10–30 min.

### 2.2 D‐112 after traumatic hemorrhage at the US Army Institute of surgical research

#### 2.2.1 Animals and experimental design

Sprague–Dawley male rats (Charles River, Wilmington, MA) were shipped at 8 weeks of age (225–250 g) and acclimated for 1 week before transmitter surgery. At the end of experimental procedures, animals were 11–12 weeks and weighed 300–450 g. Environmental conditions met the Institute for Laboratory Animal Research (ILAR) Guide recommendations. Room temperature was maintained at 20–26°C with lights on from 0600 to 1800 h. Food (LabDiet 5001 Rodent Diet, St. Louis, MO) and water were provided ad libitum.

Research was conducted in compliance with the Animal Welfare Act, the implementing Animal Welfare Regulations, and the principles of the Guide for the Care and Use of Laboratory Animals, National Research Council. Research conducted in this study was approved by the facility's Institutional Animal Care and Use Committee.

For this study, we used a nonblinded randomized block approach, where there was no intent to compare results from the 37% and 50% hemorrhages; effects of D‐112 were evaluated independently at each level of hemorrhage. Within each hemorrhage group, rats were assigned randomly to receive either D‐112 or vehicle. There were four experimental groups, and all groups had extremity trauma: (1) 37% hemorrhage with vehicle (lactated Ringer's; LR) injection (*n* = 10); (2) 37% hemorrhage with D‐112 injection (*n* = 10); (3) 50% hemorrhage with vehicle injection (*n* = 8); and (4) 50% hemorrhage with D‐112 injection (*n* = 11).

#### 2.2.2 Transmitter implantation surgery

Telemetry transmitters were used to measure mean arterial pressure (MAP) and heart rate (HR). The transmitter surgery procedure described previously by Klemcke et al. (2021) was followed. Briefly, anesthesia was induced with 3% isoflurane (Forane, Baxter Healthcare Corporation, Deerfield, IL) in 100% oxygen and was subsequently maintained throughout the procedure with 1%–3% isoflurane in oxygen. All surgeries were conducted under aseptic conditions. After anesthesia induction, rats were injected with sustained‐release buprenorphine, SR (1.2 mg/kg, subcutaneous, sc; Zoo Pharm, Fort Collins, CO), to provide postsurgical pain relief. A 1‐inch incision was made along the linea alba to open the abdomen. The HD‐S10 radiotelemetry transmitter (Data Sciences International (DSI), St. Paul, MN) catheter was inserted into the dorsal aorta at a site 1 cm anterior to the iliac bifurcation and was secured with skin adhesive (Vetbond). The transmitter portion was then secured to the abdominal muscle and the muscle and skin were closed with suture. Animals were allowed to recover in plastic cages with a layer of clean towels and water access for 3 h prior to transferring to their original home cage.

#### 2.2.3 Carotid artery catheterization

**FIGURE 1:**
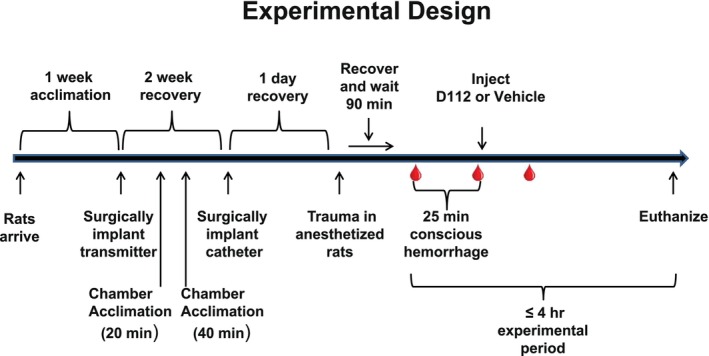
Experimental timeline. Note that the scale of designated time periods does not accurately reflect the actual time periods but is illustrative only. Blood drops represent the time at which arterial blood samples were taken (i.e., at the start of hemorrhage, at the end of hemorrhage, and at 30 min after the hemorrhage was complete).

Figure 1 shows the experimental timeline. Two weeks after transmitter surgery, rats were anesthetized again to undergo carotid artery catheter surgery as described in detail previously (Klemcke et al., 2008, 2021). Briefly, PU‐25 tubing (Braintree Scientific, Braintree, MA) was inserted into the left common carotid artery and exteriorized in the dorsal neck region. During the surgical placement of catheters, sterile heparin sodium in 0.9% sodium chloride solution (Baxter Healthcare Corporation, 2B0944, 2 USP per mL) was used as a filling solution. Then, heparin (Instech, USP‐HS‐020‐1‐NJP‐50, 20 USP/mL) sodium in saline was used in the locking solution and was sealed with a sterile stainless‐steel wire plug. The carotid catheter was used for both blood withdrawal and intra‐arterial administration of vehicle or D‐112.

#### 2.2.4 Extremity trauma

Approximately 24 h after catheterization, rats were anesthetized a third time with 3% isoflurane for a total of 10 min to attach extension tubing and undergo extremity trauma as previously described (Klemcke et al., 2023; Xiang et al., 2018). Briefly, a soft tissue injury was conducted on the right hind leg by clamping the gastrocnemius and semimembranosus muscles for 30 s with an angled Kelly clamp. Immediately after, the fibula was fractured in a controlled fashion via external pressure. Tubing extension (Micro‐Renathane, Braintree Scientific, treated previously with TDMAC) was attached to the PU‐25 tubing protruding from the dorsal neck. Rats were returned to a recovery cage, kept warm under a heat lamp, and allowed to recover from anesthesia for 90 min.

#### 2.2.5 Hemorrhage and D‐112 testing

90‐min after extremity trauma, the conscious rat was placed into a whole‐body plethysmography chamber, and the extension tubing was exteriorized and connected to a 1‐mL syringe containing sterile saline. Whole‐body plethysmography was conducted using DSI's Halcyon Whole‐Body Plethysmography (WBP)‐Rat (Model 601–1428‐001) set at a bias flow of 1.5 L/min and associated FinePointe WBP Controller and FinePointe software as described previously [18]. Respiratory measures were collected and recorded over 1‐min intervals. Primary respiratory measures were respiration rate (RR; breaths/min) and tidal volume (TV; mL; volume of air inspired or expired with each normal breath). Minute ventilation (MV; mL/min; volume of new air moved into the respiratory passages each minute) was calculated as the product of RR × TV.

HR and MAP were detected via an HD‐S10 DSI PhysioTel Hybrid Digital transmitter. The MAP and HR signals were transmitted to the receiver, which then sent the signals to the acquisition system, where the signal was digitized with the associated software (Ponemah Data Acquisition System), as described previously [18]. MAP and HR were acquired at a sampling rate of 500 Hz and were logged every minute.

Baseline cardiovascular and respiration measures were recorded for 5 min immediately before the initiation of hemorrhage. Thirty‐seven (37%) or fifty (50%) percent of each rat's calculated blood volume was withdrawn from the conscious rat via the carotid catheter extension following this rate schedule: 8%/min during the first 5 min, 4%/min during the next 10 min, and 2%/min during the last 10 min of the hemorrhage (Klemcke et al., 2008, 2021). The total hemorrhage time was therefore 25 min. The average blood volume value of 6.93 (0.45) mL/100 g body weight, determined in a separate group of 17 rats in our laboratory, was used to calculate the volume of blood to be removed from each rat based on its body weight on the day of the experiment.

**FIGURE 2:**
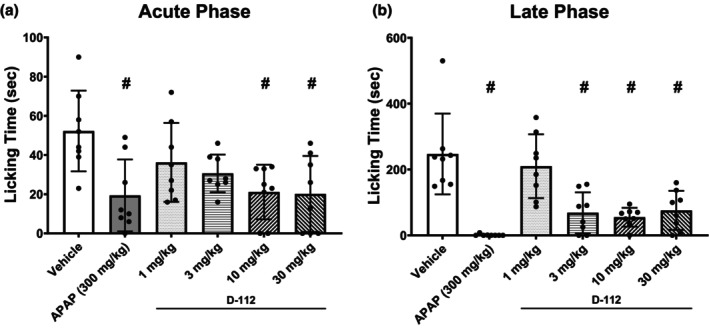
Effects of D‐112 on formalin‐induced inflammatory pain in rats. Acute phase (0–5 min) of the formalin test (a) and late phase (10–30 min) (b) of the formalin test in rats treated with vehicle, APAP, and D‐112. Each treatment column shows the mean and SD bars obtained in *n* = 8. Dunnett's test with *p* < 0.05 is indicated with a hash mark (#).

When the 25‐min hemorrhage was completed, D‐112 (50 mg/kg) and the remaining saline flush (added to ensure complete drug delivery; the two volumes add to 1 mL) were slowly injected over 75 s. Alternatively, a comparable volume of vehicle and saline flush was injected into control rats. The dose of D‐112 (50 mg/kg) selected was based on the data shown in Figure 2, discussions with Kalyra Pharmaceuticals, and preliminary screening studies in our laboratory (data not shown). We used the highest dose of D‐112 tolerated in our laboratory for testing in the ET and HEM model (50 mg/kg) to maximize chances of observing adverse effects on cardiorespiratory variables. Cardiovascular and respiratory measures were recorded for the next 215 min (240 min from hemorrhage start) or until death. After hemorrhage, rats that either: (1) displayed a MAP less than 25 mmHg, or (2) reached 4‐h post‐initiation of hemorrhage were euthanized with an intravascular injection of sodium pentobarbital (150 mg/kg body weight).

Approximately 300 μL of the first (Start Hemorrhage) and last blood samples of the hemorrhage (End Hemorrhage), and a ~300 μL sample taken 30 min post‐hemorrhage [Time (*t*) = 55 min], were analyzed using the i‐STAT Handheld Blood Analyzer (Abbott, Princeton, NJ). CG4 and CHEM8 cartridges provided base excess, lactate, arterial oxygen saturation (SaO_2_), arterial oxygen pressure (PaO_2_), hemoglobin (Hb), hematocrit, bicarbonate (HCO_3_), potassium, and glucose concentration measurements. Blood oxygen content was calculated as: Oxygen Content (mL = dL) = (Hb × 1.34 × SaO_2_) + (0.003 × PaO_2_) (Klemcke et al., 2021; Torres et al., 2004).

#### 2.2.6 Data analysis and statistics

Cardiovascular and respiratory data were recorded throughout baseline, hemorrhage, and post hemorrhage periods. During data analysis, these 1‐min observations were averaged for each rat over 5 min periods that corresponded with the above‐noted phases of the hemorrhage: (1) 5‐min baseline before hemorrhage, (2) min 21 through 25 of hemorrhage, (3) min 26 through 30 post hemorrhage, (4) min 31 through 35 post hemorrhage, and (5) every 30 min subsequently for 5‐min periods (i.e., at 61–65; 91–95; 121–125; 151–155; 181–185; 211–215; and 236–240 min) after initiation of the hemorrhage. Values for each time interval were then averaged across all rats within a treatment. Survival time was recorded from the start of hemorrhage to either the time of death, when the death criteria were met (MAP of <25 mmHg), or at the end of the 240 min experiment.

Data were analyzed using GraphPad Prism 10.5.0 (DotMatics, Boston, MA). Formalin test data were analyzed with a one‐way ANOVA (acute phase) or Kruskal–Wallis test (late phase) followed by a two‐sided Dunnett's (acute phase) or Dunn's (late phase) multiple comparison test to compare values for drugs/doses against that of the vehicle control. For most data, a mixed‐effects repeated‐measures model using a compound symmetry covariance matrix and Geisser–Greenhouse correction was conducted to analyze differences between treatments (vehicle or D‐112) and times (11 levels for cardiorespiratory data, three levels for blood chemistry data). Multiple mean comparisons within treatments across time were conducted using two‐sided Dunnett's multiple comparisons tests to compare against the immediate post‐hemorrhage value (*t* = 25 min). To compare the two treatments at each time point, two‐sided Šídák's multiple comparisons tests were used. When rats survived the complete 4 h and were euthanized, the true survival time is unknown; such data are said to be “censored.” These survival data with censored observations were analyzed using the two‐sided Log‐rank (Mantel‐Cox) test for determining differences among survivor functions. Survival times were analyzed using a two‐sided Mann–Whitney nonparametric test (37% hemorrhage) or a two‐sided *t*‐test (50% hemorrhage). Data are presented as arithmetic means ± standard deviation (SD). All tests were performed at a false positive error rate of *α* = 0.05.

## 3 RESULTS

### 3.1 Formalin test

In the formalin test, control rats spent an average of 52 s licking the injected paw in the acute phase (Figure 2a) and 247 s in the late phase (Figure 2b). Treatment effects were present in both early (*p* = 0.003) and late (*p* < 0.001) phases. Specifically, APAP reduced the time spent licking the injected paw in both the acute (*p* = 0.002) and the late phase (*p* < 0.001) of the formalin test. D‐112 produced inhibition of the pain response during both the acute and late phases of formalin‐induced licking, demonstrating its analgesic effect.

### 3.2 37% hemorrhage

All rats in the 37% hemorrhage group (*n* = 10) that were treated with vehicle survived, and 80% of the rats that were treated with D‐112 (*n* = 10) survived; there was no difference (*p* = 0.146) in the survival percentages. Survival times also did not differ (*p* = 0.474) between groups (vehicle, 240 (0) min; D‐112, 214 (54) min).

**FIGURE 3:**
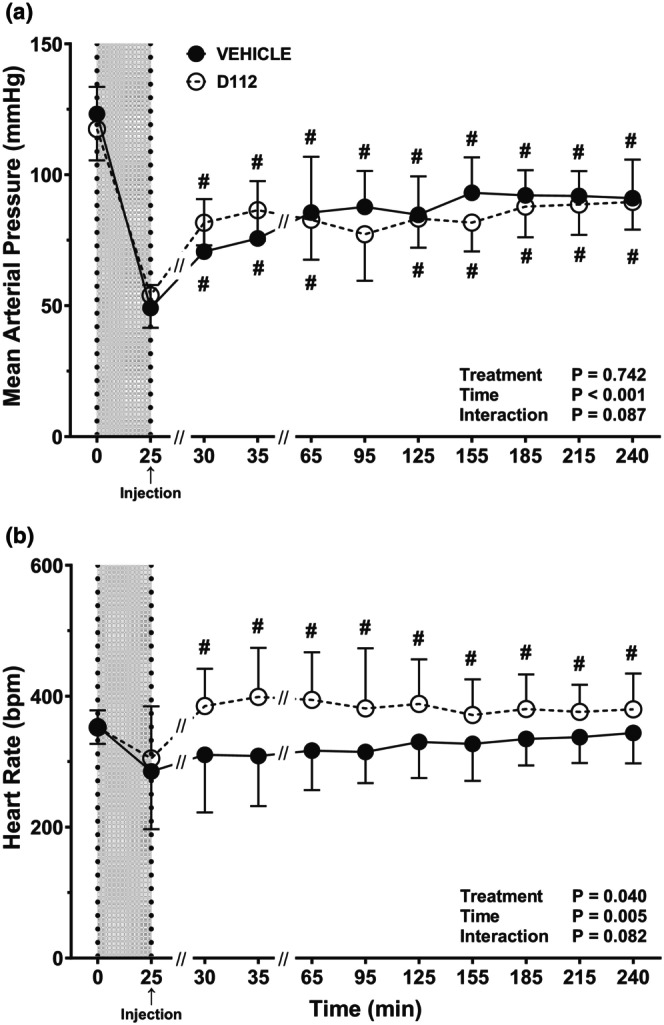
Mean arterial pressure (MAP; panel a), and heart rate (HR; panel b) in rats following extremity trauma and hemorrhage of 37% estimated blood volume. Vehicle (LR) group is *n* = 10 and D‐112 (50 mg/kg) group is *n* = 10. *N* sizes at each time point: For vehicle group, *n* = 10 at each time point and for D‐112 group, *n* = 10 for *t* = 0–95, *n* = 8 for *t* = 125–240. The gray area indicates hemorrhage time. Vehicle (LR) or D‐112 (50 mg/kg) injection occurred immediately post‐hemorrhage, at *t* = 25 min. Mean and SD bars are displayed at each time point, with the (#) symbol indicating Dunnett's test *p* ≤ 0.05 from the post‐hem/pre‐injection value (*t* = 25 min) within treatment group. There were no differences between treatment groups at any time point.

MAP decreased after 37% hemorrhage in vehicle and D‐112 groups with no difference between groups (*t* = 25 min; *p* = 0.987). Within the vehicle‐treated rats, compared with MAP at 25 min, MAP was elevated post‐hemorrhage (*t* = 30 through *t* = 240 min; Figure 3a). Compared with MAP at 25 min, MAP also increased post‐hemorrhage in the D‐112‐treated rats, except for one time point (*t* = 95 min). Overall, there was no treatment effect on MAP, and values for vehicle rats were not different from those for D‐112 rats at any time point (Figure 3a). HR was not affected by hemorrhage in either vehicle or D‐112 groups (i.e., no difference between *t* = 0 and *t* = 25 min; Figure 3b). HR remained constant post‐hemorrhage in the vehicle group but increased in the D‐112 group (*t* = 30 through *t* = 240 min compared to *t* = 25 min). There was an overall treatment effect when comparing vehicle versus D‐112 rats, as seen in Figure 3b (*p* = 0.040). However, there were no differences (*p* ≥ 0.161) between vehicle and D‐112 groups at any time point.

**FIGURE 4:**
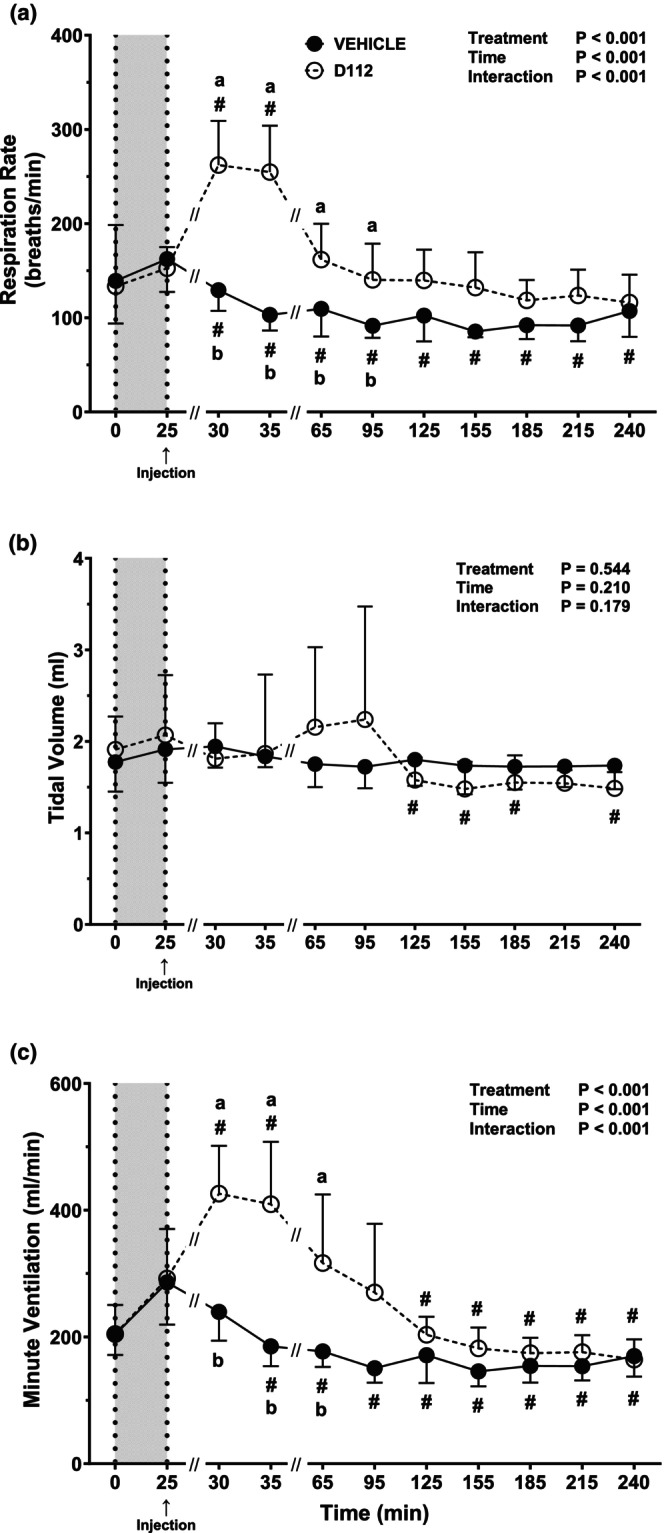
Respiratory rate (RR; panel a), tidal volume (TV; panel b), and minute ventilation (MV; panel c) in rats following extremity trauma and hemorrhage of 37% estimated blood volume injected with either vehicle or D‐112. *N* sizes at each time point are the same as for CV data. Different letters (A, B) indicate a treatment difference (vehicle versus D‐112) at that time using Šídák's test *p* < 0.05. See Figure 3 legend for additional description of the graphs.

For both vehicle and D‐112 groups, RR did not change with 37% hemorrhage (*p* ≥ 0.80 between *t* = 0 and *t* = 25 min; Figure 4a). RR decreased post‐hemorrhage in vehicle rats and remained low until the end of the experiment (*t* = 240 min). In contrast, RR increased transiently (*t* = 30 and 35 min; *p* = 0.001 at each time point) post‐hemorrhage in D‐112 rats. RR was higher 5–70 min post‐hemorrhage (*t* = 30 through *t* = 95 min) in D‐112 compared to vehicle‐treated rats (Figure 4a). TV was unchanged by hemorrhage and remained constant post‐hemorrhage in vehicle rats, but D‐112 rats decreased slightly at *t* = 125 min and onward. There was no treatment effect on TV when comparing vehicle to D‐112 rats (Figure 4b). MV increased due to hemorrhage in vehicle‐ and D‐112‐treated rats (Figure 4c) with no difference between groups. In vehicle‐treated rats, MV decreased 10 min post‐hemorrhage (*t* = 35 min) and remained lower post‐hemorrhage until the end of the experiment (*t* = 240 min). However, MV transiently increased after D‐112 injection (*t* = 30 and 35 min) before decreasing below post‐hemorrhage levels at *t* = 125 min and onward. MV was elevated in the D‐112 group compared to the vehicle group at 5–40 min post‐hemorrhage (*t* = 30 through *t* = 65 min; *p* ≤ 0.029).

As expected with reduced tissue perfusion and anaerobic metabolism in hemorrhage, lactate increased (Time: *F* = 13.88, *p* = 0.003; Treatment: *F* = 0.057, *p* = 0.814; Interaction: *F* = 1.71, *p* = 0.205) approximately 8.5‐fold, while base excess decreased (Time: *F* = 16.42, *p* < 0.0001; Treatment: *F* = 0.156, *p* = 0.697; Interaction: *F* = 1.694, *p* = 0.204) during 37% hemorrhage, within the vehicle group (Table 1). 30 min post‐hemorrhage within the vehicle group, lactate decreased (*p* = 0.001) relative to the end of hemorrhage, whereas base excess increased (*p* = 0.021), and oxygen content remained constant compared to the end of hemorrhage. Within the D‐112 group, neither lactate, base excess, nor oxygen content differed from the end of hemorrhage to 30 min post‐hemorrhage (*p* ≥ 0.770). There were no differences between treatment groups (vehicle vs. D‐112) at baseline, end of hemorrhage, or 30 min post‐hemorrhage for any arterial blood measures in Table 1.

**TABLE 1:** Oxygen content and metabolic indices associated with trauma, hemorrhage, and D‐112 treatment in rats.

37% hemorrhage				
Measure	Treatment	Time		
		Start hemorrhage	End hemorrhage	30 min post‐hemorrhage
		(*n* = 10/group)	(*n* = 9–10/group)	(*n* = 8–10/group)
Lactate (nM)	Vehicle	0.5 (0.4)	4.3 (2.3)	3.0 (1.7)^#^
	D‐112	0.4 (0.1)	3.3 (1.8)	4.7 (5.4)
O_2_ Content (ml/dL)	Vehicle	17.6 (1.7)	13.1 (1.8)	12.6 (1.4)
	D‐112	18.2 (1.9)	14.5 (1.9)	14.0 (0.8)
Base excess (nM)	Vehicle	6.9 (2.3)	0.4 (3.5)	2.8 (2.3)^#^
	D‐112	7.5 (3.1)	1.4 (2.8)	−0.4 (8.9)

50% hemorrhage				
Measure	Treatment	Time		
		Start hemorrhage	End hemorrhage	30 min post‐hemorrhage
		(*n* = 8–11/group)	(*n* = 8–10/group)	(*n* = 6–11/group)
Lactate (nM)	Vehicle	0.4 (0.1)	10.7 (2.1)	9.3 (2.9)
	D‐112	0.4 (0.2)	9.8 (2.5)	10.9 (5.5)
O_2_ Content (ml/dL)	Vehicle	17.5 (2.7)	12.4 (2.4)	11.1 (1.3)
	D‐112	18.1 (2.0)	12.0 (1.7)	12.2 (1.1)
Base excess (nM)	Vehicle	8.0 (1.4)	−8.3 (2.7)	−6.5 (5.9)
	D‐112	8.3 (1.4)	−8.9 (3.8)	−7.7 (6.1)

*Note*: Values are means (SD). Sample sizes vary due to either technical difficulties in obtaining or processing the blood sample, or to deaths following 50% hemorrhage. Data were analyzed by mixed effects model using GraphPad PRISM. Means comparisons at each time point were analyzed using Šídák's multiple comparisons tests. There were no treatment differences in any measure at any time point. The 30 min post‐hemorrhage means were compared to End Hemorrhage within treatment using an unadjusted pairwise test, with the symbol (#) signifying *p* ≤ 0.05.

At 30 min post‐hemorrhage, hematocrit (*p* = 0.028) and hemoglobin (*p* = 0.032) levels were higher in D‐112 compared to vehicle‐treated rats, but no other arterial blood measures (including HCO_3_^−^, glucose, PCO_2_, potassium, and pH) differed between treatment groups (Table S1).

### 3.3 50% hemorrhage

**FIGURE 5:**
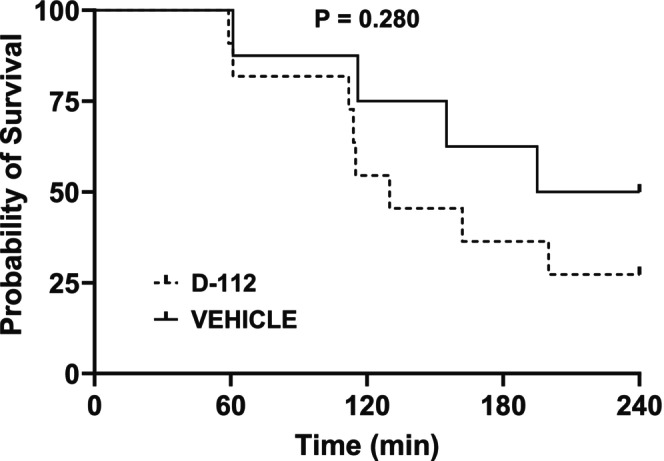
Survival in rats following extremity trauma and hemorrhage of 50% estimated blood volume. Kaplan–Meier survival curve for vehicle (LR) and D‐112 (50 mg/kg) groups; *n* = 8 for vehicle group, *n* = 11 for D‐112 group. Censored data (survivors) reflect the absence of a true survival time as these rats were euthanized at 4 h after initiation of hemorrhage. Data were analyzed using GraphPad Prism associated Kaplan–Meier procedure for estimating survivor functions and log‐rank test for determining differences among survivor functions.

In the 50% hemorrhage groups, the survival percentages at 240 min were 50.0% for rats treated with vehicle (*n* = 8), and 27.3% for rats that were treated with D‐112 (*n* = 11) (Figure 5). There was no survival curve treatment effect of D‐112 (*p* = 0.280). Survival times also did not differ (*p* = 0.306) between vehicle (186 (69) min) and D‐112 (152 (69) min) groups.

**FIGURE 6:**
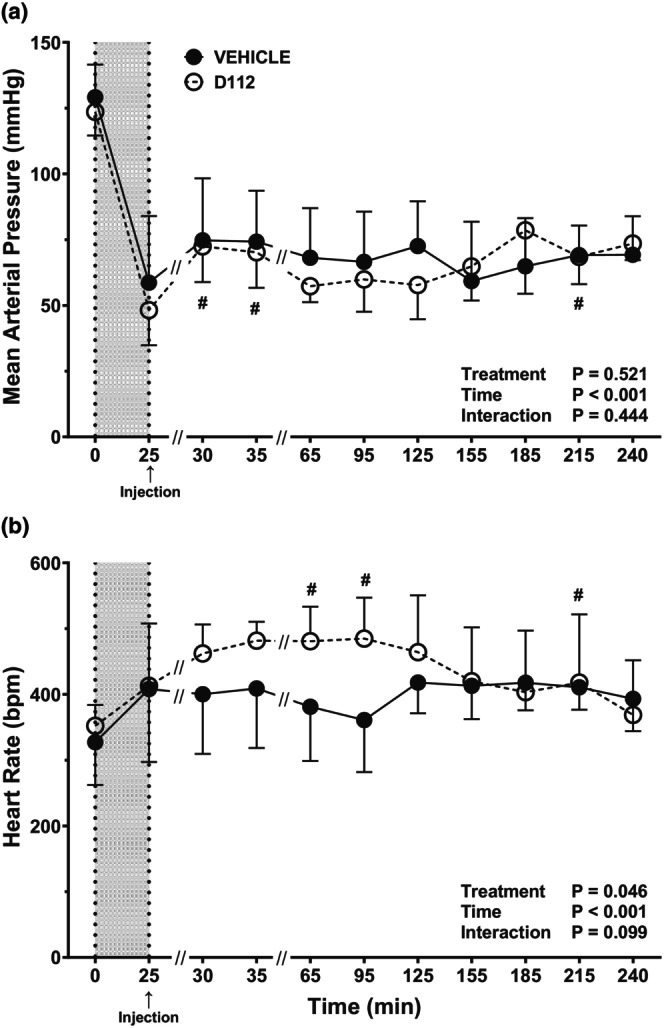
Mean arterial pressure (MAP; panel a) and heart rate (HR; panel b) in rats following extremity trauma and hemorrhage of 50% estimated blood volume injected with either vehicle or D‐112. Initial sample sizes were *n* = 8 for the vehicle group, *n* = 11 for the D‐112 group. *N* sizes at each time point: For the vehicle group, *n* = 8 for times 0–35, *n* = 7 for *t* = 65 and *t* = 95, *n* = 6 for *t* = 125 and *t* = 155, *n* = 5 for *t* = 185, and *n* = 4 for *t* = 215 and *t* = 240. For the D‐112 group, *n* = 11 for times 0–35, *n* = 9 for *t* = 65 and *t* = 95, *n* = 6 for *t* = 125, *n* = 5 for *t* = 155, *n* = 4 for *t* = 185, *n* = 3 for *t* = 215, and *n* = 2 for *t* = 240. There were no differences between treatment groups at any time point. See Figure 3 legend for additional description of the graphs.

For both vehicle and D‐112 groups, MAP was significantly lower at *t* = 25 min, the end of hemorrhage, than at the beginning of hemorrhage (*p* < 0.001), but there was no difference between treatment groups (Figure 6a). In vehicle rats, MAP remained at low levels following hemorrhage compared to baseline. Rats in the D‐112 group had a MAP that was significantly higher at *t* = 30 and *t* = 35 min compared to *t* = 25 min (*p* < 0.001 and *p* = 0.001, respectively) but MAP subsequently returned to *t* = 25 min levels. There was no D‐112 treatment effect on MAP compared to vehicle at any time point. HR was not affected by 50% hemorrhage in either vehicle or D‐112 groups (Figure 6b). There was no change in HR in vehicle rats throughout the experimental period. For the D‐112 group, HR was higher at both *t* = 65 and *t* = 95 min than at *t* = 25 min (*p* = 0.007 and *p* = 0.009, respectively). Despite an absence of treatment significance at any individual time point, the overall treatment effect (*p* = 0.046) across all time periods argues for a D‐112‐associated increase in heart rate during the 30–95 min time frame.

**FIGURE 7:**
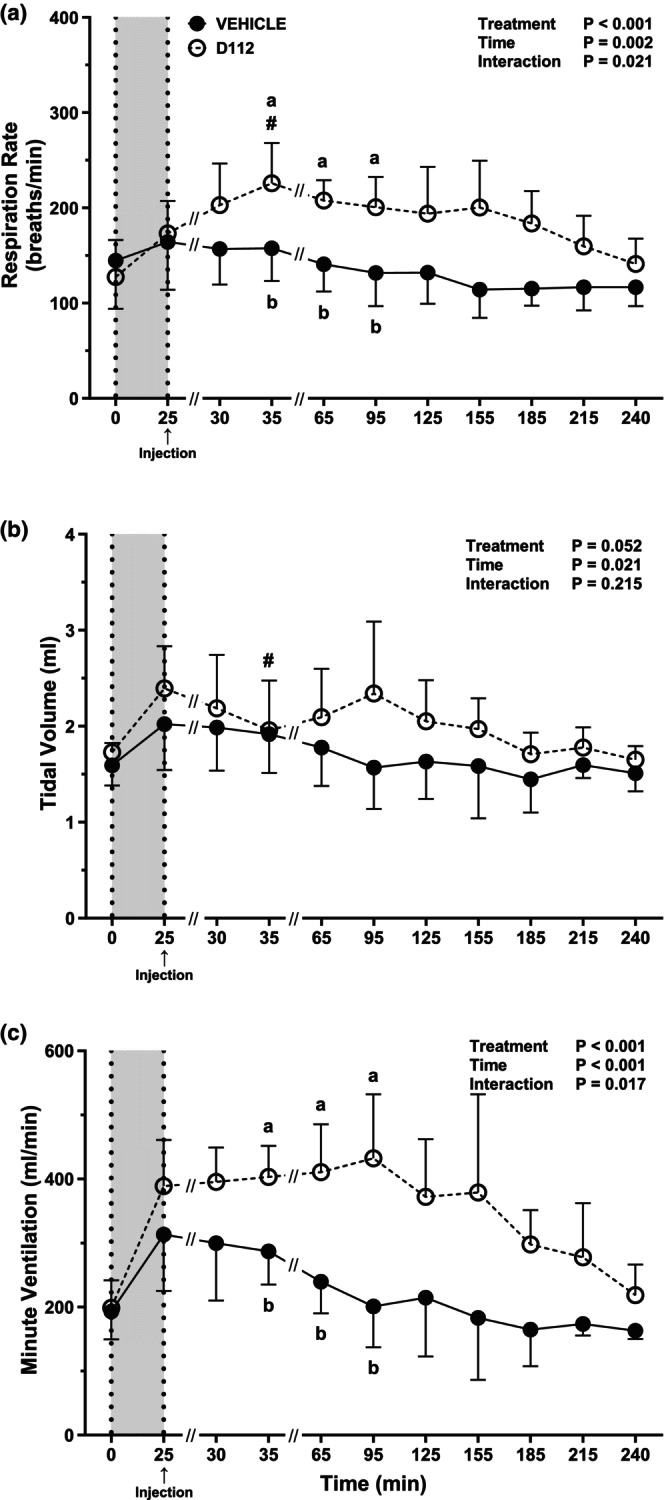
Respiratory rate (RR; panel a), tidal volume (TV; panel b), and minute ventilation (MV; panel c) in rats following extremity trauma and hemorrhage of 50% estimated blood volume injected with vehicle or D‐112. *N* sizes at each time point are the same as for 50% hemorrhage CV data. Different letters (A, B) indicate a treatment difference (vehicle vs. D‐112) using Šídák's test *p* < 0.05. The (#) symbol indicates Dunnett's test *p* ≤ 0.05 from the post‐hem/pre‐injection value (*t* = 25 min) within the treatment group.

After 50% hemorrhage, RR remained constant for the vehicle group until the end of the study (*t* = 240 min) (Figure 7a). For the D‐112 group, RR was transiently higher at *t* = 35 min compared to *t* = 25 min (*p* = 0.007). D‐112 increased RR (*p* < 0.001). Specifically, RR differed between D‐112 and vehicle at *t* = 35 min (*p* = 0.013), *t* = 65 min (*p* = 0.004), and *t* = 95 min (*p* = 0.016). Across these three time points, vehicle RR increased an average of 48% compared to D‐112 (Figure 7a). There was no change in TV with hemorrhage in the vehicle group (Figure 7b), but TV was higher in the D‐112 group at the end of hemorrhage (*t* = 25 min) than at the beginning of hemorrhage (*t* = 0 min; *p* = 0.007). TV remained constant after the end of hemorrhage in the vehicle group but decreased transiently in the D‐112 group at *t* = 35 min compared to *t* = 25 min (*p* = 0.016). Overall, there was no effect of D‐112 on TV compared to vehicle at any time. MV was higher after hemorrhage in both groups (*p* ≤ 0.02; Figure 7c). There were no significant changes in MV across time within each group post‐hemorrhage. However, the D‐112 group had a higher MV at *t* = 35, 65, and 95 min (*p* ≤ 0.002) compared with the vehicle group.

In both groups, lactate (Time: *F* = 77.47, *p* = 0.0001; Treatment: *F* = 0.733, *p* = 0.790; Interaction: *F* = 0.807, *p* = 0.455) increased approximately 25‐fold while base excess (Time: *F* = 131.4, *p* < 0.0001; Treatment: *F* = 0.146, *p* = 0.707; Interaction: *F* = 0.213, *p* = 0.810) and oxygen content (Time: *F* = 173.1, *p* < 0.0001; Treatment: *F* = 0.294, *p* = 0.595; Interaction: *F* = 1.252, *p* = 0.303) decreased after hemorrhage (Table 1). For the D‐112 group, glucose decreased at 30 min post‐hemorrhage compared to the end of hemorrhage (*p* < 0.009) (Table S1). For the vehicle group, potassium increased at 30 min post‐hemorrhage compared to the end of hemorrhage (*p* < 0.009) (Table S1). There were no differences between treatment groups (vehicle vs. D‐112) at baseline, at the end of hemorrhage, or 30 min post‐hemorrhage for any of the arterial blood measures in Table 1 and Table S1 in the 50% hemorrhage rats.

## 4 DISCUSSION

For this study, our goal was to evaluate the effects of a novel APAP analog, D‐112, on cardiorespiratory and survival variables after hemorrhage and in the presence of ET. The APAP analogs developed by Kalyra Pharmaceuticals, including D‐112, offer a promising approach to treat pain without the liver toxicity that often accompanies high doses of APAP. There were two major findings in this study. The first is that D‐112 transiently increased RR, which subsequently drove an increase in MV. Secondly, however, D‐112 did not negatively affect post‐hemorrhage survival or cardiovascular and respiratory compensatory responses to either moderate (37%) or severe (50%) traumatic hemorrhage.

We have previously first determined the analgesic efficacy of currently available battlefield analgesics (i.e., ketamine and opioids) (Xiang et al., 2018) and then the effects of these analgesic doses on physiological compensatory mechanisms to traumatic hemorrhage (Klemcke et al., 2021; Marshall‐Lipiec et al., 2025). In this study, the formalin test was first used to determine D‐112 analgesic efficacy. The formalin test is a useful model to determine the analgesic efficacy of novel drugs because it models both acute and inflammatory pain (Abbott et al., 1995; Dubuisson & Dennis, 1977; Hoffmann et al., 2022; McNamara et al., 2007; Stucky et al., 2009). Like APAP, D‐112 decreased formalin‐induced nocifensive responses, paw licking (Figure 2). While there are no published studies using D‐112, another APAP analog developed by Kalyra Pharmaceuticals that has been studied is KP‐1199. KP‐1199 (60 mg/kg) reduced thermal allodynia in a model of thermal injury in rats where the same dose of APAP (60 mg/kg) was ineffective (Reddoch‐Cardenas et al., 2021). Additionally, KP‐1199 showed no risk of platelet activation, and it did not inhibit coagulation in whole blood samples, suggesting it could be a promising alternative to APAP in cases where blood loss and blood clotting are of concern (Reddoch‐Cardenas et al., 2021). Preliminary results indicate that D‐112 also has analgesic effects when tested in a thermal injury model in male rats (Fowler et al., 2017). D‐112 also has the potential to be used as a safer alternative to APAP since it does not produce the hepatotoxic metabolite NAPQI (Reddoch‐Cardenas et al., 2021). This suggests that D‐112 is a worthy candidate for further testing in a battlefield‐relevant model of traumatic hemorrhage.

To understand the effects of an analgesic dose of D‐112 administered after hemorrhage as would be encountered in a pre‐hospital environment, we gave D‐112 (50 mg/kg) after 37% and 50% hemorrhage. The major difference between the D‐112 and vehicle‐treated groups was a transient increase in RR following both hemorrhage levels, which drove an increase in MV. There are limited studies of APAP effects on systemic respiratory function (i.e., RR and TV). Sewell et al. showed that APAP caused respiratory depression but at very high doses (500–1000 mg/kg) (Sewell et al., 1984). APAP has some action within the respiratory tract, as it has been previously shown to induce oxidative stress throughout the respiratory tract when administered at high doses (100–200 mg/kg) (Smith et al., 2016). Cytochrome P450 2E1 is also expressed in peripheral lung tissue and its expression can increase after APAP exposure, leading to APAP‐protein adducts, activation of endoplasmic reticulum stress, and proinflammatory signaling (Sandoval et al., 2019). Pro‐oxidants and transient receptor potential cation channel subfamily A member 1 (TRPA1), a polymodal sensor and cation channel, activation can change lung function and lead to decreased RR (Taylor‐Clark & Undem, 2011). Airway irritation at high doses of APAP (200 mg/kg) could also be due to NAPQI activation of the TRPA1 channel (Nassini et al., 2010). However, many of the previously reported adverse effects of APAP on respiration are due to the production of NAPQI (Kennon‐McGill & McGill, 2018) and this metabolite is not present after D‐112 administration by design. We also observed that the transient increase in RR caused by D‐112 was not sufficient to change O_2_ content or PCO_2_ in arterial blood measures, perhaps because the change in RR was not contemporaneous with our blood sampling. Therefore, it is unlikely that D‐112 activated TRP channels not only because it does not metabolize to produce NAPQI but also because it produced an increase in RR rather than respiratory depression as seen with APAP. The mechanism of action for the increase in RR is unknown but appears to be distinct from the general effects of APAP and may be tied to the novel pharmacology of D‐112.

One effect of D‐112 on cardiovascular physiology was an overall increase in HR. Following drug injection, HR increased over time within the D‐112‐treated groups under both hemorrhage conditions, while there was no further increase in HR over post‐hemorrhage levels in the vehicle‐treated groups. Although there were no statistically significant differences in HR between vehicle‐ and D‐112‐treated groups at any single timepoint, there was an overall treatment effect. In both moderate and severe hemorrhage, MAP measurements in vehicle‐ and D‐112‐treated groups closely resembled each other with no treatment effects noted. Although there are no studies examining the cardiovascular effects of D‐112, previous data on APAP offer mixed information about its effect on cardiovascular variables. For example, APAP increased cardiac output and MAP in sheep but had no effect when tested in rabbits; the increase in cardiac output in sheep was not produced by an increase in HR, which is not consistent with our results (Leshnower et al., 2006). In febrile critically ill human patients, APAP significantly lowered MAP and HR (Schell‐Chaple et al., 2017). Intravenous APAP has also been shown to cause hypotension in critically ill patients, increasing their mortality (van der Horst et al., 2020). Transient hypotension can also develop in healthy individuals after intravenous APAP (Chiam et al., 2016). APAP caused a transient decrease in MAP in anesthetized rats that was attenuated by pretreatment with a potassium channel (Kv7) inhibitor, linopirdine (van der Horst et al., 2020). The APAP metabolite NAPQI enhances the activity of Kv7 channels (van der Horst et al., 2020). It is possible that D‐112 did not produce hypotension in the current study because this APAP analog was specifically developed to circumvent the production of NAPQI, which may mediate the cardiovascular effects of APAP.

Importantly, there were no differences in either survival percentages or survival times between vehicle‐ and D‐112‐treated groups following either moderate or severe hemorrhage. The conscious hemorrhage model we utilized included ET to recapitulate the injuries that a soldier might withstand in the battlefield (Xiang et al., 2018); that is, hemorrhage does not occur in the absence of tissue injury in trauma victims and so must be a part of relevant hemorrhage models. The presence of ET itself decreases survival after severe hemorrhage (Klemcke et al., 2023). 37% hemorrhage is a moderate hemorrhage that activates compensatory mechanisms that can normalize cardiovascular variables over time. Thus, as seen here and in previous studies, there was a 100% survival in the 37% hemorrhage vehicle group (Klemcke et al., 2021, 2023; Marshall‐Lipiec et al., 2025). 50% hemorrhage is a severe hemorrhage where compensatory mechanisms may not be enough to recover physiological levels of HR or MAP; therefore, leading to death in a substantial percentage of animals. In this study, there was no statistical difference identified between survival curves in either hemorrhage severity, suggesting that D‐112 may be safe even in a rodent model in which physiological function is already severely compromised by hemorrhage. It should be noted, however, that the study was not powered to detect differences in survival time due to the large variability in survival times that we have previously observed using this rat model (Klemcke et al., 2021; Marshall‐Lipiec et al., 2025). We therefore performed a post hoc power analysis using the survival probabilities measured herein after 50% hemorrhage. In order to achieve a power of 0.8 at a probability of 0.05, the sample sizes would have to be 161 rats per treatment. Such a large number strongly suggests the probability that, for the conditions of this study, our conclusion of no difference among the treatment groups is accurate and indicates the futility of continuing the study in an attempt to achieve significant differences among treatments.

Previous studies have delineated limitations that accompany the ET and hemorrhage model in part due to a necessity to adhere to ethical experimental designs that minimize animal suffering while standardizing procedures. Some of these limitations include the facts that (1) trauma and hemorrhage did not occur at the same time; (2) isoflurane was used during ET; (3) the hemorrhage was controlled; and (4) no resuscitation was used (Klemcke et al., 2021, 2023). Another limitation is that we only used male rats for our study. Males make up the majority of trauma patients in both military and civilian casualties (Shackelford et al., 2021; Wild et al., 2020) as well as civilian non‐battlefield patients (Oyeniyi et al., 2017). For example, a recent retrospective study from 2014 to 2021 showed that 93% of U.S. military fatalities were male (Kotwal et al., 2023), and 97% of the patients documented in the Department of Defense Trauma Registry are male (Shackelford et al., 2021). Males are therefore the appropriate target population for this initial study. Another limitation is that we did not obtain antinociception data in the ET model. Even though D‐112 analgesic efficacy has been tested in various pain models (incision, neuropathic, formalin; data not published), it would be useful to understand its effects following ET. Furthermore, we only tested one dose of D‐112 following traumatic hemorrhage. The dose of D‐112 that was chosen was the highest dose that was tolerated by animals in preliminary testing in our laboratory (unpublished observations) and it was also a dose that was higher than the doses tested in the formalin test. We chose to test a higher dose in the ET and hemorrhage model to maximize the opportunity to observe adverse effects of D‐112, perhaps unmasking effects that might not be seen at lower doses. Along these lines, we have previously estimated that drug concentrations attained after injection may have reached 1.27‐ (37% hemorrhage) and 1.52‐fold (50% hemorrhage) higher than would have occurred in non‐hemorrhaged rats (Klemcke et al., 2021), further creating a “worst case scenario” that might unmask any potential adverse effects. It should be noted that doses of analgesics administered to trauma patients are also not adjusted for estimated blood volume loss.

## 5 CONCLUSION

In conclusion, trauma and hemorrhage are typically accompanied by pain. For both civilian and military patients, it is important to treat pain with medications that do not interfere with compensatory responses of the cardiovascular and respiratory systems that attempt to maintain homeostasis in the prehospital setting (Fisher et al., 2022; Gausche‐Hill et al., 2014; Petz et al., 2015). For this study, we used a previously established model that combines ET with one of two different types of hemorrhage (moderate and severe) to test the effects of the novel APAP analogue D‐112 on cardiorespiratory responses and survival (Klemcke et al., 2021). Here we show that D‐112 had analgesic efficacy in the formalin test like APAP. We also observed that, at the dose tested, D‐112 did not alter cardiovascular compensatory mechanisms. Even though there were modest and transient effects on respiration, there was no indication of respiratory depression, and the effects observed did not translate into blood oxygen changes or adverse effects on survival. Thus, if our rat model can be indicative of similar effects in humans, D‐112 may be considered a safe analgesic candidate to treat pain in the battlefield even among those individuals that have or are at risk for developing hemorrhage.

## AUTHOR CONTRIBUTIONS

MMP analyzed data and prepared the manuscript. KLR analyzed data, interpreted the results, reviewed and edited the manuscript. MLC, CMR, BSC, and SLP conducted the experiments and collected data. LEG performed statistical analyses. HGK and CHL designed this research, procured funding, interpreted the results, reviewed and edited the manuscript. KB and CH conducted formalin testing, collecting and analyzing the data. All authors read and approved the final manuscript.

## FUNDING INFORMATION

This work was supported by the US Army Combat Casualty Care Research Program at the US Army Medical Research and Development Command, Proposal CCCRP D_007_2017.

## CONFLICT OF INTEREST STATEMENT

Kevin Bunker founded Kalyra Pharmaceuticals. Chad Hopkins was an employee of Kalyra Pharmaceuticals. They provided data on formalin testing but were not involved in the testing of D‐112 in the traumatic hemorrhage models.

## ETHICS STATEMENT

Research was conducted in compliance with Animal Welfare Act, the implementing Animal Welfare regulations, and the principles of the Guide for the Care and Use of Laboratory Animals. The Institutional Animal Care and Use Committee approved all research conducted in this study. The facility where this research was conducted is fully accredited by the AAALAC International.

## DISCLAIMER

The opinions or assertions contained in this article are those of the authors and do not reflect the official policy or position of the U.S. Army Futures Command, Department of the Army, Department of Defense, or the U.S. Government.

## Supporting information


Table S1.

## Data Availability

The data underlying this article will be shared on reasonable request to the corresponding author.
