# Supplementary material for: Effects of a novel acetaminophen analog on cardiorespiratory compensatory responses and survival in a male rat model of traumatic hemorrhage
Source: Physiol Rep. 2025 Nov 16;13(22):e70619. doi: 10.14814/phy2.70619 (PMC12620408; doi:10.14814/phy2.70619)
Supplement: Supplementary file 1 — Table S1. [file PHY2-13-e70619-s001.docx]

**Supplemental Table 1**. Arterial blood gases and metabolic indices associated with trauma, hemorrhage and D-112 treatment in rats.

| **37% Hemorrhage** | | | | |
| --- | --- | --- | --- | --- |
| **Measure** | **Treatment** | **Time** | | |
|  |  | **Start Hemorrhage**  **(n=10/group)** | **End Hemorrhage**  **(n=9-10/group)** | **30 min Post-hemorrhage**  **(n=9-10/group)** |
| HCO_3_ (mM) | Vehicle | 30.8 (1.9) | 23.0 (2.9) | 26.2 (2.4) ^#^ |
|  | D-112 | 31.1 (2.8) | 23.7 (3.1) | 23.1 (7.3) |
| Glucose (mg/dl) | Vehicle | 144.7 (13.6) | 268.1 (41.8) | 257.6 (84.0) |
|  | D-112 | 152.7 (23.6) | 260.7 (63.5) | 193.3 (30.0) ^#^ |
| Hematocrit (%) | Vehicle | 39.0 (3.7) | 28.2 (4.2) | 27.4 (3.2) |
|  | D-112 | 40.2 (4.3) | 31.5 (4.0) | 31.3 (2.7) ^a^ |
| Hemoglobin (g/dL) | Vehicle | 13.3 (1.3) | 9.6 (1.4) | 9.3 (1.1) |
|  | D-112 | 13.7 (1.5) | 10.7 (1.4) | 10.6 (0.9) ^a^ |
| pCO_2_ (mmHg) | Vehicle | 42.7 (1.4) | 27.0 (4.2) | 34.7 (4.2) ^#^ |
|  | D-112 | 42.2 (2.8) | 27.7 (5.3) | 28.4 (8.3) |
| Potassium (mM) | Vehicle | 3.8 (0.4) | 4.3 (0.3) | 3.7 (0.4) ^#^ |
|  | D-112 | 3.9 (0.3) | 4.1 (0.3) | 4.1 (0.4) |
| pH | Vehicle | 7.47 (0.02) | 7.54 (0.06) | 7.49 (0.03) |
|  | D-112 | 7.47 (0.02) | 7.55 (0.05) | 7.48 (0.10) |
| **50% Hemorrhage** | | | | |
| **Measure** | **Treatment** | **Time** | | |
|  |  | **Start Hemorrhage**  **(n=8-11/group)** | **End Hemorrhage**  **(n=6-10/group)** | **30 min Post-hemorrhage**  **(n=6-11/group)** |
| HCO_3_ (mM) | Vehicle | 31.6 (1.4) | 15.1 (2.5) | 17.6 (5.7) |
|  | D-112 | 32.1 (1.4) | 14.7 (3.4) | 16.1 (5.1) |
| Glucose (mg/dl) | Vehicle | 155.0 (18.1) | 370.1 (76.9) | 357.3 (90.7) |
|  | D-112 | 147.6 (14.3) | 374.5 (58.1) | 237.2 (117.8) ^#^ |
| Hematocrit (%) | Vehicle | 38.9 (6.0) | 25.8 (4.9) | 25.9 (6.0) |
|  | D-112 | 40.2 (4.6) | 25.7 (3.9) | 29.0 (6.2) |
| Hemoglobin (g/dL) | Vehicle | 13.2 (2.1) | 8.8 (1.7) | 8.8 (2.0) |
|  | D-112 | 13.7 (1.6) | 8.7 (1.3) | 9.8 (2.1) |
| pCO_2_ (mmHg) | Vehicle | 43.7 (2.3) | 19.0 (3.9) | 23.6 (7.9) |
|  | D-112 | 44.7 (2.7) | 20.2 (3.7) | 22.7 (6.5) |
| Potassium (mM) | Vehicle | 3.8 (0.4) | 5.1 (0.8) | 4.0 (0.4) ^#^ |
|  | D-112 | 4.0 (0.3) | 4.7 (0.6) | 5.1 (1.8) |
| pH | Vehicle | 7.47 (0.01) | 7.47 (0.06) | 7.45 (0.03) |
|  | D-112 | 7.46 (0.02) | 7.47 (0.04) | 7.38 (0.20) |

Values are means (SD). Sample sizes vary due to either technical difficulties in obtaining or processing the blood sample, or to deaths following 50% hemorrhage. Data were analyzed by a mixed effects model using GraphPad PRISM. Means comparisons within treatments at each time point were analyzed using Sidak’s multiple comparisons tests, with the letter (a) indicating P ≤ 0.05. The 30-minute post-hemorrhage means were compared to End Hemorrhage within treatment using an unadjusted pairwise test, with the symbol (#) signifying P ≤ 0.05.
